# Genetic Analysis Using an Isogenic Mating Pair of *Aspergillus fumigatus* Identifies Azole Resistance Genes and Lack of *MAT* Locus’s Role in Virulence

**DOI:** 10.1371/journal.ppat.1004834

**Published:** 2015-04-24

**Authors:** Liliana Losada, Janyce A. Sugui, Michael A. Eckhaus, Yun C. Chang, Stephanie Mounaud, Abigail Figat, Vinita Joardar, Suman B. Pakala, Suchitra Pakala, Pratap Venepally, Natalie Fedorova, William C. Nierman, Kyung J. Kwon-Chung

**Affiliations:** 1 J. Craig Venter Institute, Rockville, Maryland, United States of America; 2 Molecular Microbiology Section, Laboratory of Clinical Infectious Diseases, National Institute of Allergy and Infectious Diseases, National Institutes of Health, Bethesda, Maryland, United States of America; 3 Division of Veterinary Resources, Office of Research Services, National Institutes of Health, Bethesda, Maryland, United States of America; 4 National Cancer Institute, National Institutes of Health, Bethesda, Maryland, United States of America; Carnegie Mellon University, UNITED STATES

## Abstract

Invasive aspergillosis (IA) due to *Aspergillus fumigatus* is a major cause of mortality in immunocompromised patients. The discovery of highly fertile strains of *A*. *fumigatus* opened the possibility to merge classical and contemporary genetics to address key questions about this pathogen. The merger involves sexual recombination, selection of desired traits, and genomics to identify any associated loci. We constructed a highly fertile isogenic pair of *A*. *fumigatus* strains with opposite mating types and used them to investigate whether mating type is associated with virulence and to find the genetic loci involved in azole resistance. The pair was made isogenic by 9 successive backcross cycles of the foundational strain AFB62 (*MAT1-1*) with a highly fertile (*MAT1-2*) progeny. Genome sequencing showed that the F_9_ *MAT1-2* progeny was essentially identical to the AFB62. The survival curves of animals infected with either strain in three different animal models showed no significant difference, suggesting that virulence in *A*. *fumigatus* was not associated with mating type. We then employed a relatively inexpensive, yet highly powerful strategy to identify genomic loci associated with azole resistance. We used traditional *in vitro* drug selection accompanied by classical sexual crosses of azole-sensitive with resistant isogenic strains. The offspring were plated under varying drug concentrations and pools of resulting colonies were analyzed by whole genome sequencing. We found that variants in 5 genes contributed to azole resistance, including mutations in *erg11A* (*cyp51A*), as well as multi-drug transporters, *erg25*, and in HMG-CoA reductase. The results demonstrated that with minimal investment into the sequencing of three pools from a cross of interest, the variation(s) that contribute any phenotype can be identified with nucleotide resolution. This approach can be applied to multiple areas of interest in *A*. *fumigatus* or other heterothallic pathogens, especially for virulence associated traits.

## Author Summary

Invasive aspergillosis (IA) caused by *Aspergillus fumigatus* is increasing due to medical interventions that suppress the ability of patients’ immune systems to control infections. These invasive lung infections are difficult to diagnose and consequently treatment is frequently not started promptly. Some controversy surrounds the role of mating type in virulence of *A*. *fumigatus* and the emergence of azole resistant strains has posed difficult challenges for clinical management of IA. We generated nearly identical *A*. *fumigatus* strains with opposite mating types that allowed us to test whether different mating types have different virulence profiles. We found no difference in virulence in three different animal models, which suggests that mating type does not influence virulence. We also took advantage of the essentially identical genomes of both strains to apply classical genetic approaches combined with genomics technologies to identify *A*. *fumigatus* genes that contribute to azole resistance. We performed genetic crosses of azole sensitive with azole resistant strains and analyzed the resistance status and genome composition of the offspring. Using this approach we cataloged several genes that were not previously associated with azole resistance. This information will be valuable for finding ways to manage azole resistance in IA patients.

## Introduction

In spite of the significant progress made in diagnosis and treatment of patients with invasive aspergillosis (IA), the number of patients that succumb to the infection is still unacceptably high. Early diagnosis and treatment that can eradicate the fungus without further compromising the patients’ health remain as the most pressing challenges faced in the treatment of IA [1]. In the pathobiology of *A*. *fumigatus*, the predominant etiological species of IA, remarkable progress has been made since sequencing of its genome [2,3]. Moreover, the recently discovered heterothallic sexual cycle in the species [4] offers an unique opportunity to study genes relevant for virulence or drug resistance using genetic recombination [5].

Following the description of the sexual cycle in *A*. *fumigatus* [4], we described a pair of *A*. *fumigatus* supermaters that produce unusually large numbers of cleistothecia containing viable progeny in 4 weeks [5]. Even though the supermater pair can be used in an array of recombinational analysis, studies focusing on the association of genomic sequences and phenotype can be better addressed when the genomes of two mating strains are identical except for their mating type and sequences of interest. For instance, in the investigation of mating type and virulence in *A*. *fumigatus* it is critical to use isogenic strains to normalize the genetic background and target differences specific to the phenotype. Generation of isogenic strains and their use for genetic analysis enabled extensive progress in the molecular pathobiology of *Cryptococcus neoformans* including the role of mating type in fungal virulence [6–9]. In *A*. *fumigatus*, some studies suggested a predominance of *MAT1-1* strains among cases of IA and a more severe virulence phenotype for *MAT1-1* compared to *MAT1-2* isolates in the larvae of *Galleria mellonella* [10,11]. Comparisons in these studies, however, have been made between *MAT1-1* and *MAT1-2* isolates of unknown genetic background from clinical and/or environmental sources. Because of the many factors associated with the pathobiology of *A*. *fumigatus*, it is difficult to specifically target the contribution of mating type in the virulence of such isolates with heterogeneous genetic background.

Increasingly important issues in clinical management of IA are the general limitation in treatment options—only three classes of drugs are available—and the ever-increasing problem of drug resistance [12]. Systemic aspergillosis infections are treated with amphotericin B, azoles [13], or caspofungin. The low efficacy of the available antifungal drugs requires a long duration of treatment that frequently leads to resistance for azoles and caspofungin [12,14]. Antifungals exploit differences between mammalian and fungal cells to kill the fungal organisms without adverse effects on the host. Drugs used to treat fungal infections target only two differences between human and fungal cells: the presence of ergosterol in fungal cell membranes and glucans in their cell walls [13]. Azole antifungals (e.g. itraconazole, posaconazole, voriconazole, fluconazole) inhibit an enzymatic step in the ergosterol biosynthetic pathway. Prevalent and well-studied resistance mechanisms in bacteria, fungi, and parasites include target modification and efflux from the cell. However, there remains an urgent need to understand the broad range of genes encoded in the genomes of fungal pathogens that participate in the resistance to the clinically therapeutic antifungals employed in treating infections.

Whole-genome sequencing of resistant strains was recently used to identify novel drug-resistance mechanisms in *Escherichia coli* [15], *Candida glabrata* [16], and *Plasmodium falciparum* [17], among others. *A*. *fumigatus* is a haploid fungus and colonies grown in vitro have average 5–7 nuclei in each hyphal cell [18] which makes the species very amenable to sequencing-based screen as it reduces the complications of diploid organism. Camps *et al*. used sexual crosses between clinically-isolated azole-resistant and sensitive strains of *Aspergillus fumigatus* to follow mutations that conferred resistance [19]. They identified mutations in the *hapE* gene, and demonstrated that sexual crossing is a powerful mechanism to confirm the role of specific mutations in resistance.

In this study we generated an isogenic pair of *A*. *fumigatus* with identical genomes except for the mating locus and the absence of a single 28 Kb region in one mating type. The isogenic pair was used in virulence and drug resistance studies. Virulence studies in animal models indicated that the mating type of *A*. *fumigatus* is not associated with virulence phenotypes. We then employed azole sensitive and resistant variants of the isogenic strains in classical genetic crosses to follow mutations that were linked with the resistance phenotype in order to more clearly elucidate which mutations actually confer resistance. The isogenic pair, AFB62 and AFB62F9, represents a powerful tool to study the pathobiology of *A*. *fumigatus*.

## Results

### Generation of an *Aspergillus fumigatus* isogenic mating pair by successive backcrossing

The *A*. *fumigatus* strains AFB62 (*MAT1-1*), a clinical isolate, and AFIR928 (*MAT1-2*), an environmental isolate, were chosen as starting parental strains because of their high fertility [5]. The *MAT1-1* parent strain, AFB62, was chosen as the foundational strain for backcrossing. A highly fertile *MAT1-2* progeny from a cross between AFB62 and AFIR928 was backcrossed with AFB62 and the resulting progeny of *MAT1-2* mating type was backcrossed again with AFB62. After 4 generations of backcrossing, the genomes of an AFB62F4 (*MAT1-2)* strain and the foundational strain AFB62 were compared by comparative genome hybridizations (CGH). Of the genes in the AF293 microarray, the results showed that AFB62F4 and AFB62 were nearly isogenic with only a 7-gene difference between the strains (S1 Table). In contrast, the two starting parental strains had 86 absent or divergent genes [5]. In order to reach a more isogenic state, back-crossings were continued for seven additional generations (F_11_). A highly fertile ninth generation progeny, AFB62F9, was selected for use throughout the rest of this study since the fertility declined significantly after F_9_. The ninth generation isogenic pair AFB62F9 and AFB62 was able to produce viable ascospores in 4 weeks and the ascospore viability was on average 13%, which is comparable to 17% found for the supermaters AFI928 and AFB62.

To examine if the isogenic pair progeny exhibited Mendelian inheritance, we generated a mutant of AFB62 producing brown conidia by the deletion of the gene *abr2* (Afu2g17530) [5] and a mutant of AFB62F9 producing white (albino) conidia by deletion of the *alb1/pksP* (Afu2g17600) gene. Mating of these two strains generated progeny that produced white, brown, and green conidia (S1 Fig). Since the *abr2* and *alb1* loci are both located on the chromosome 2, the presence of green conidia denotes reconstitution of the wild type genotype by crossing-over between the mutated loci. Independent assortment of the mating-type alleles located on chromosome 3 and the conidial color genes on chromosome 2 was confirmed by the observation of near 1:1 ratio of *MAT1-1* to *MAT1-2* in the progeny and 2.5–7% frequency of the wild type recombinants producing green colored conidia (S1 Fig). These findings showed that fertility and recombination of the isogenic pair were not significantly different from the supermater pair, AFB62 and AFIR928 (5–10% green conidial recombinants) [5], confirming the usefulness of the isogenic pair for genetic analysis.

### Genome comparison of AFB62 and AFB62F9

The genomes of AFB62 and AFB62F9 were sequenced to determine the extent of their isogenicity. The AFB62 genome consisted of 27,882,634 bp and was assembled into 895 contigs ranging from 502 to 351,863 bp, with N_50_ of 78,619 bp. Comparisons between AFB62 and the genomes of *A*. *fumigatus* reference strains AF293 and A1163, indicated that AFB62 is more closely related to A1163 with many fewer SNPs (51,765 SNP and 30,263 SNP, respectively). The majority of the genomic differences between AFB62 and the two reference strains were concentrated in the sub-telomeric regions of the chromosomes or in genomic islands, which was similar to the previous observations between different *A*. *fumigatus* isolates [20]. Over 94% (8,789 genes) of AFB62 genes had homologs in AF293 and A1163. Most of the AFB62-unique genes (371 out of 493) had no assigned function (i.e. hypothetical), encoded for proteins with generic transmembrane domains, or belonged to one of 26 different GO terms with no significant enrichment in any one term (S2 Table). It is important to note, however, that the quality of the AFB62 genome assembly is significantly lower than either of the two reference genomes which can artificially inflate the total number of predicted open reading frames, and consequently, overestimate the number of unique genes.

**Fig 1:**
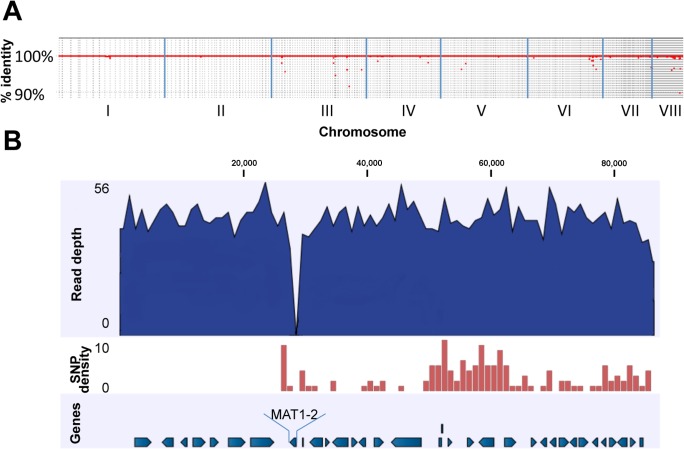
Genomic comparison of AFB62F9 and AFB62. (A) Whole genome coverage plot using AFB62F9 reads mapped to the AFB62 genome showing percent identity (y-axis) per chromosome (x-axis). The red line and dots represents regions of the AFB62 genome covered by AFB62F9 reads at greater than 90% identity. (B) Close-up of mapped reads to the region surrounding the *MAT1-2* locus in AFB62. Numbers on top are coordinates on contig 677 (coordinates 1495670–1582078 on chromosome III). Top track in dark blue: average coverage of the genome was 48X with a maximum of 56 read coverage. The *MAT1-2* locus in AFB62F9 had no mapped reads so the coverage drops to 0X (~27 Kb in this figure). Second track: SNP density per 1,000 bp is depicted as a red histogram. Maximum SNP/Kb was 10. Note the sharp increase surrounding the *MAT1-1* locus and extending in the 3’ direction for an additional ~230Kb not depicted in this figure. Bottom track: annotated protein-coding genes in the AFB62 assembly. Direction of arrows depicts the coding strand.

The AFB62F9 sequencing reads were mapped to the AFB62 reference genome in order to identify differences and similarities between the two isogenic strains. Over 99.5% of the sequencing reads were mapped to the AFB62 reference for which almost all the residues (99.91%) were covered by AFB62F9 reads, showing that AFB62F9 is identical to AFB62 except for the *MAT1-2* instead of *MAT1-1* mating locus (Fig 1A) and the absence of a 28 Kb region located about 200 Kb away from the mating locus of AFB62 on chromosome 3 (S2 Fig). A close inspection of the AFB62F9 reads mapped to the chromosomal region encompassing the mating locus and the 28 Kb locus (~290 Kb) showed a high number of point mutations (e.g. SNP) in the AFB62F9 genome compared to the AFB62 genome. A total of 413 SNP were located in this region, which was almost half of all identified point mutations that differentiated the isogenic strains (849 total). The SNP density for this region was 14.2 SNP /10,000 bp, which was much larger than the rest of the genome with an average of 0.05 SNP/10,000 bp. This SNP density is similar to the SNP density obtained when different strains of *A*. *fumigatus* are compared to each other [21]. Taken together, the high SNP density and the absence of a 28 Kb fragment show that the *MAT1-2* locus in AFB62F9 was inherited from the AFIR928 parent through recombination of this relatively large fragment (Figs 1B and S2). A non-related SNP-dense region ~400 Kb was also found on chromosome 6 (AF293 coordinates 645,096 to 1,045,729) containing 287 SNP (7.2 SNP/10,000 bp), suggesting that this region too was inherited as a single block from the AFIR928 parent. This region encoded a sister-chromatid segregation protein homolog (*src1*) an adenylate cyclase (*acyA*), and two transcription factors including TFIID. In AFB62F9, the promoter region of this gene had a 4 base pair deletion and a G→A substitution that could affect its transcriptional regulation. The *acyA* region in AFB62F9 contained 9 SNP including a non-synonymous substitution (P91H), which could potentially lead to different activity of this protein. Lastly, a frame shift mutation in TFIID complex could also have a significant impact in the transcriptional response of AFB62F9 compared to AFB62.

### Virulence and mating type

**Fig 2:**
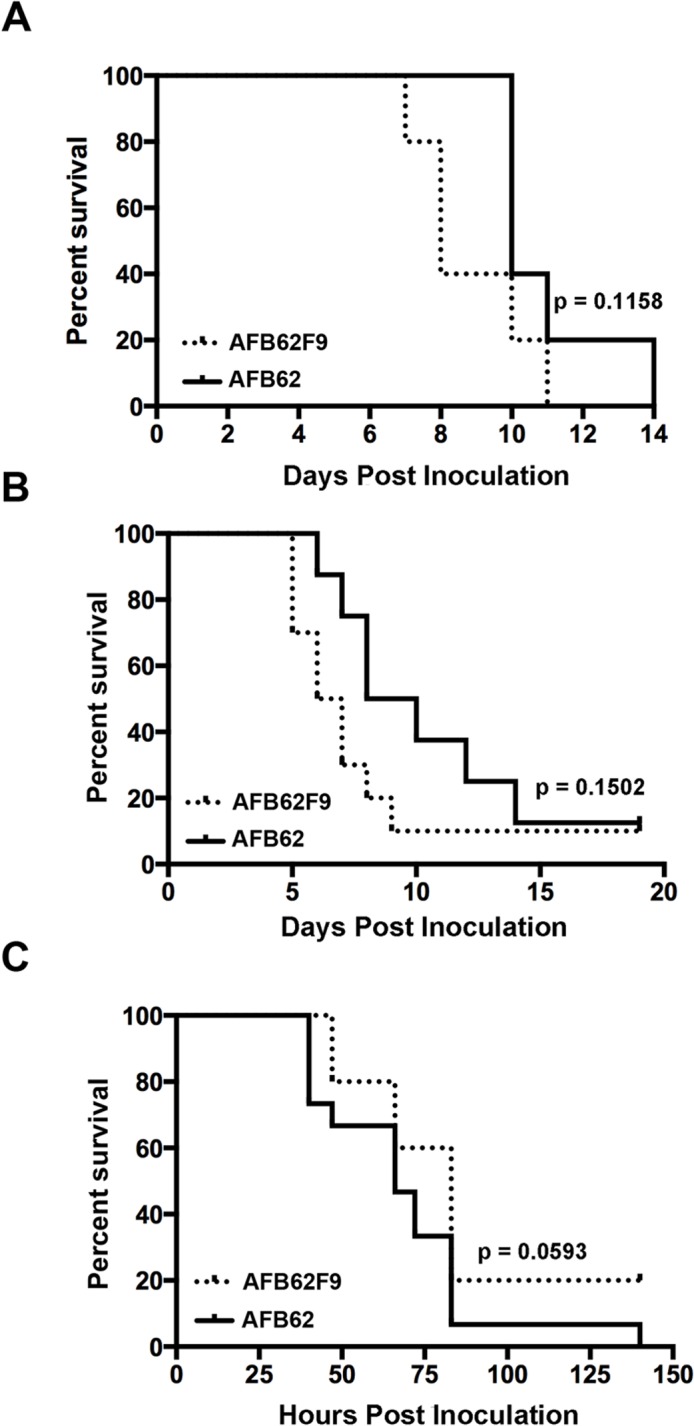
Virulence studies in murine and larvae models. Mice with chronic granulomatous disease (CGD) (A), hydrocortisone treated BALB/c mice (B) and *Galleria mellonella* larvae (C) were inoculated with *Aspergillus fumigatus MAT1-1* (AFB62) or *MAT1-2* (AFB62F9) strains. CGD (5 mice per group) and BALB/c (10 mice per group) mice received 30 μl of 3.33x10^5^ and 3.33x10^7^ conidia/ml, respectively, via pharyngeal aspiration. Larvae (15 larvae per group) were injected with 5 μl of 2x10^7^ conidia/ml. Survival data was analyzed using log rank test.

**Fig 3:**
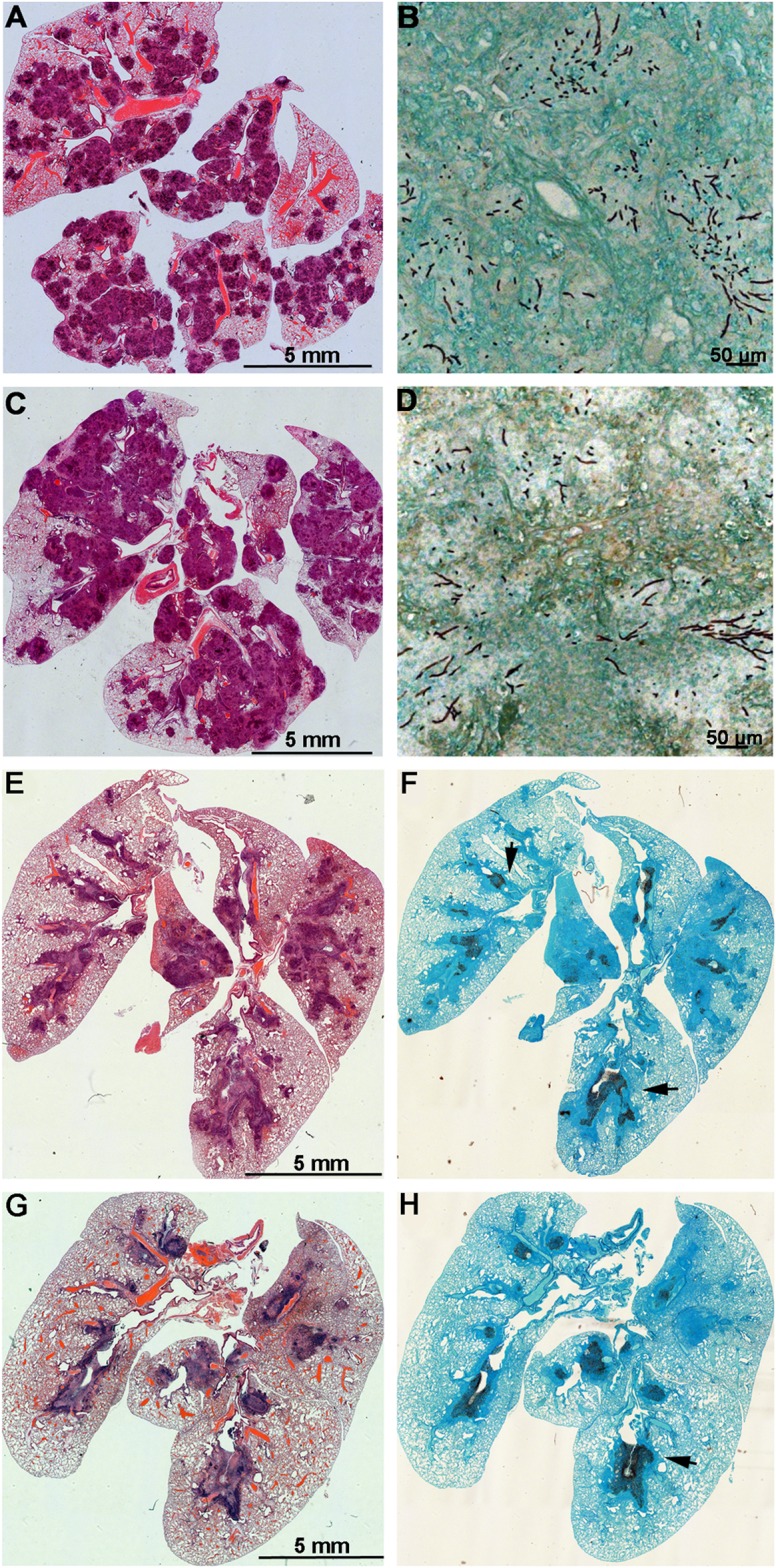
Lungs of mice with chronic granulomatous disease (CGD) or hydrocortisone-treated BALB/c mice inoculated with *Aspergillus fumigatus* isogenic strains, AFB62 and AFB62F9. (A-D) Lungs of CGD mice. (E-H) Lungs of hydrocortisone-treated BALB/c mice. (A, B, E and F) Lungs of mice inoculated with AFB62. (C, D, G and H) Lungs of mice inoculated with AFB62F9. Numerous granulomas (A and C) with extensive hyphal growth (B and D) are seen in the lungs of CGD mice at nine days post fungal inoculation. Extensive hyphal growth (arrows in F and H) is seen in the bronchial tree in lungs of hydrocortisone-treated BALB/c mice at seven days post fungal inoculation. CGD and hydrocortisone-treated BALB/c mice were inoculated with 30 μl of 3.33x10^5^ and 3.33x10^7^ conidia/ml, respectively, via pharyngeal aspiration. Sections were stained with hematoxylin and eosin (A, C, E and G) and Gomori methenamine silver (B, D, F and H).

The two parental lines, AFB62 and AFIR928, are equally virulent in mouse models and have similar virulence to other isolates such as B-5233, a reference strain of *A*. *fumigatus* [5]. However, due to their other genomic differences, it was not possible to determine whether the mating locus played a specific role in pathobiology of *A*. *fumigatus*. In this study, the virulence between AFB62 and AFB62F9 strains was first compared using CGD mice that are susceptible to *A*. *fumigatus* due to an impaired production of reactive oxygen species by the NADPH oxidase complex [22,23]. No significant difference was found between the survival curves of mice inoculated with AFB62 and that of mice inoculated with AFB62F9 suggesting these two strains possess similar virulence in CDG mice (Fig 2A). Histological sections of lungs inoculated with AFB62 and AFB62F9 showed similar lesions with micronodular bronchopneumonia affecting approximately 50–75% of the parenchyma (Fig 3A and 3C). Branching septate hyphae admixed with moderate number of neutrophils was observed in the smaller bronchioles and adjacent alveoli and moderate necrosis was observed in alveolar septae. Larger airways and bronchial epithelium were relatively spared of inflammatory infiltrate and fungal hyphae. Additional virulence assays carried out with two *MAT1-2* progeny of the 7^th^ generation confirmed the findings with the 9^th^ generation progeny.

Virulence of AFB62 and AFB62F9 was then tested in BALB/c mice immunosuppressed with hydrocortisone acetate. No significant difference was found between the survival curves of the mice inoculated with AFB62 and AFB62F9 (Fig 2B). Histology of lungs isolated 7 days post inoculation showed that both strains caused similar lesions: severe necrotizing bronchopneumonia with large numbers of septate branching hyphae with approximately 25–33% of the parenchyma being affected; prominent necrosis of the bronchial/bronchiolar epithelium; bronchial/bronchiolar lumina presented hyphae admixed with neutrophilic cell infiltrate, necrotic cells and fibrin (Fig 3E–3H).

The last model used to compare virulence between AFB62 and AFB62F9 was larvae of *Galleria mellonella*. This model has been used in several studies of virulence in *A*. *fumigatus*, including comparison between several random *MAT1-1* and *MAT1-2* strains of clinical and environmental origin [11,24–26]. Larvae inoculated with AFB62 died at the same rate as those inoculated with AFB62F9 (Fig 2C). Similar results were obtained when larvae were inoculated with five *MAT1-1* and five *MAT1-2* F_10_ progenies isolated from the cross between AFB62 and AFB62F9. The results of the virulence assays carried out in three different experimental models indicated *MAT1-1* and *MAT1-2* mating type strains in the AFB62 genomic background possess similar ability to grow invasively in lungs (Fig 3B and 3D), cause similar pathology of the disease and are equipotent in causing host death, suggesting that the *MAT* locus does not play a significant role in virulence of *A*. *fumigatus*.

### In vitro selection and analysis of azole resistance in *A*. *fumigatus*

**Fig 4:**
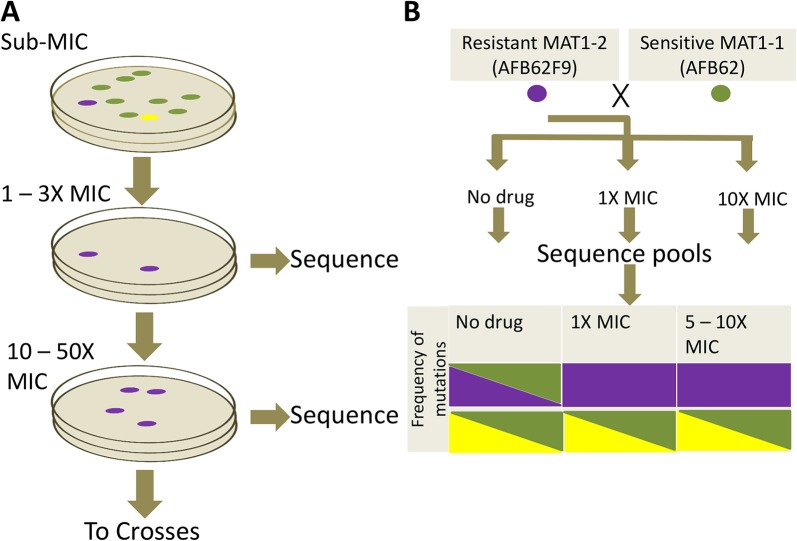
Azole resistance experimental design. (A) Sensitive AFB62 or AFB62F9 cells were incubated on solid media containing sub-MIC concentrations of each of itraconazole, voriconazole, or posaconazole. Resulting spores were plated on selective media with at least 1X MIC of each antifungal to obtain fewer than 100 colonies. Three resistant colonies (purple) from each selective plate were subsequently and independently plated on highly selective plates containing 10 – 50X MIC of each antifungal. Three highly resistant strains (purple) were selected for sequencing and for sexual crosses. (B) Each of the highly resistant isolates from (A) was crossed with the sensitive isogenic strain of the opposite mating type. Ascospores from selected crosses were grown on media with no drug, 1X MIC and 10X MIC of the drug and their DNA sequenced to identify resistance allele frequencies. Mutant alleles are depicted in purple and yellow reference alleles in green. Cells filled with a single color represent an allele frequency of 1, cells with two colors represent an equal proportion of mutant and reference alleles. The purple mutation shows the expected frequencies for an allele associated with resistance, while the yellow mutation shows an expected frequency for alleles not associated with resistance.

We next undertook a study to identify mutations in the *A*. *fumigatus* genome that could result in resistance to itraconazole, posaconazole, and voriconazole. We subjected the isogenic pair, AFB62 and AFB62F9, to increasing concentrations of each of three azoles individually in order to select for such mutations (Fig 4A). Previous studies have shown that incubation in sub-lethal concentration of antimicrobials enhances the rate of spontaneous mutations that confer resistance [27]. Thus, we first determined the minimum inhibitory concentrations (MIC) of itraconazole, voriconazole, and posaconazole for both isogenic strains using E-strips, to allow us to ‘prime’ the strains with advantageous mutations prior to selection. Both strains were susceptible to the tested azoles (S3 Table) and fell within the observed MIC for other *A*. *fumigatus* strains [28]. Each isogenic strain was incubated in sub-inhibitory concentrations (0.5 – 1X MIC) of each antifungal on MEA plates for 72 h at which point three isolates from each strain selected under each azole were plated for single spore isolation. Each of the three isolates from media containing each azole was then transferred to a new plate containing 1 – 3X the inhibitory concentration (S3 Table) for 72 h and three new resistant colonies were selected for single spore isolation. Each of the three single spores was passaged one additional time under 10X MIC to select highly resistant mutants. Interestingly, incubation at 10X MIC for each lineage resulted in lawns, suggesting that the resistance mechanisms obtained under 1 – 3X MIC were sufficient for resistance at 10X. Thus, in order to isolate lineages with mutations that contributed to higher azole resistance, we increased the drug concentration empirically by 2X until incubation allowed less than 100 colonies per plate. The highest drug concentration was at minimum 12X for voriconazole and maximum of 78X MIC for posaconazole. Again, three isolates of each lineage were picked and single isolates were selected (Fig 4A). The resistance profiles of all single isolates were confirmed using E-test.

To identify mutations and other genomic differences between the resistant strains and wild-type parents, we sequenced and compared the genomic sequences from 15 intermediate (>1X) and 21 high concentration (>12X) isolates with its parental strain (Table 1). The isolates were selected to represent all the combinations of drug and parental lineages while maximizing the read depth obtained, thus not all 72 possible isolates were sequenced. Derivative strains selected under the highest drug concentrations contained between 1 and 13 SNPs (Tables 1 and S4). Seventy-one percent (15 of 21) of the drug resistant derivatives had at least one mutation in the *erg11A* gene [29] and 38% (8 of 21) in the 3-hydroxy-3-methylglutaryl coenzyme A (HMG CoA) reductase gene (*hmg1)* (Tables 1 and S4). No azole-resistant strains had the tandem duplication in the promoter of *erg11A* commonly associated with the L398H mutation [12]. No apparent preference for resistance mechanisms was observed in either isogenic parental strain, suggesting that the differences in genetic background or mating type did not have an effect on resistance mechanisms. A detailed description of mutations associated with each azole follows.

**Table 1:** Total number of SNP of sequenced AFB62 or AFB62F9 intermediate (bold) and high level resistant isolates in itraconazole, posaconazole, or voriconazole after in vitro selection.

Strain	Total mutations	Antifungal	*erg11A* (6)	*hmg1* (4)	*erg25A* (1)	*ssc70* (1)	*ganA* (1)	ABC Transporter (1)
**AFB62I1**	**4**	Itra					1	1
*AFB62I11*	**5**	Itra	1				1	1
*AFB62I12*	**6**	Itra	1				1	1
**AFB62I2**	**5**	Itra					1	1
*AFB62I21*	**5**	Itra	1				1	1
*AFB62I31*	**8**	Itra	1				1	1
**AFB62F9I1**	**2**	Itra						
*AFB62F9I11*	**3**	Itra			1			
**AFB62F9I2**	**5**	Itra				1		
*AFB62F9I22*	**10**	Itra				1		
**AFB62F9I3**	**2**	Itra				1		
*AFB62F9I31*	**5**	Itra	1			1		
**AFB62P1**	**9**	Posa	1				1	1
*AFB62P11*	**17**	Posa	1				1	1
**AFB62P2**	**6**	Posa	1				1	1
*AFB62P21*	**7**	Posa	1				1	1
**AFB62P3**	**6**	Posa	1				1	1
*AFB62P31*	**7**	Posa	1				1	1
**AFB62F9P1**	**1**	Posa						
*AFB62F9P13*	**8**	Posa	1				1	1
**AFB62F9P2**	**2**	Posa						
*AFB62F9P23*	**6**	Posa	1				1	1
*AFB62F9P33*	**6**	Posa	1				1	1
**AFB62V1**	**5**	Vori		1			1	1
*AFB62V13*	**7**	Vori		1			1	1
**AFB62V2**	**5**	Vori		1			1	1
*AFB62V21*	**11**	Vori	1	2			1	1
**AFB62V3**	**4**	Vori		1			1	1
*AFB62V31*	**7**	Vori	1	1			1	1
*AFB62V33*	**10**	Vori	1	1			1	1
**AFB62F9V1**	**3**	Vori		1				
*AFB62F9V11*	**8**	Vori	1	1				
**AFB62F9V2**	**2**	Vori		1				
*AFB62F9V21*	**4**	Vori		1				
*AFB62F9V31*	**8**	Vori		1				
*AFB62F9V32*	**3**	Vori		1				

Number of mutations in relevant genes *erg11A*, *erg25A*, *hmg1*, *ssc70*, *ganA*, and the ABC transporter (AFUA_92.m00226) are presented. Strains in **bold** were selected under intermediate drug concentrations, strain in *italics* were direct derivatives of strains in bold selected at high drug concentrations. Numbers in parentheses refer to the number of individual SNPs identified in each gene. Details on the specific mutations are found in S4 Table. Itra—itraconazole, Posa—posaconazole, Vori—voriconazole.

#### Itraconazole resistance

Resistance to itraconazole was explained by mutations in *erg11A* (*cyp51A)*, *erg25*, *ssc70*, a multi-drug transporter (Afu4g14760), and a transcriptional activator *ganA* (Afu3g12400). High-level itraconazole resistant strains had the G54R mutation (2 of 7 strains) in *ERG11* or synonymous mutations (3 of 7 strains) in the *erg11A* gene. The other 2 strains had either a single mutation in the HXHH motif of *ERG25*, which is involved in lanosterol synthesis [30], or a mutation that would result in a non-synonymous substitution of an arginine to cytosine in the nucleotide exchange factor domain of SSC70, a stress response chaperone [31]. None of the intermediate itraconazole resistant-strains had mutations in *erg11A*, but instead had mutations in a gene coding an ABC multidrug transporter (Afu4g14760) and GanA, a transcriptional regulator involved in stress response and sporulation. Both types of mutations were retained in the high-level resistance isolates, suggesting they might be important for resistance to itraconazole.

#### Voriconazole resistance

Voriconazole resistance was mediated by mutations in *erg11A* and *hmg1*. Two out of 8 high voriconazole resistant strains harbored the Y72F mutation in the *erg11A* gene and two others had synonymous mutations in the coding sequence of *erg11A* (S4 Table). *hmg1* mutations were present in all the voriconazole resistant strains, both high and intermediate, where 80% of strains carried non-synonymous mutations at one of three different codons within the sterol sensing domain [32]. Interestingly, only voriconazole-resistant strains carried mutations in this gene, suggesting that its role in azole resistance is limited and specific to voriconazole among these three azoles.

#### Posaconazole resistance

Both parental strains had the same MIC, however, after incubation in sub-MIC concentrations each strain displayed differential resistance to posaconazole. Consistently, the AFB62 strain had higher resistance than the AFB62F9 strain (S3 Table). Each of the three AFB62 intermediate drug resistance isolates had common mutation G54R in the *erg11A* gene along with mutations in transporters (Afu4g14760 and Afu2g10530) and the transcriptional regulator *ganA* (Tables 1 and S4). However, the two F9 posaconazole intermediate-resistant strains shared a single mutation in a gene coding for a lipophilic binding protein (Afu7g06760) and no mutations in the common resistance mechanisms. However, the G54R *erg11A* mutation was observed in all of the highest level posaconazole resistant strains, including the AFB62F9 derivatives. These results suggest that posaconazole strongly selects for the G54R in the *erg11A* gene and that at least one alternate intermediate resistance mechanism exists. In addition to *erg11A* mutations, all AFB62 posaconazole resistant strains (intermediate or high) and the high resistance AFB62F9 strains had mutations in *ganA* and two multi-drug transporters (Afu4g14760 and Afu2g10530). Interestingly, the mutations within the lipophilic binding protein in the AFB62F9 lineages were lost under high drug selection, suggesting that reversion and novel mutations occurred under high drug selection. Alternatively, because the AFB62F9 lineages were consistently selected under lower drug concentrations than the AFB62 strains, it is possible that the observed intermediate resistance was transitory.

#### Non-coding mutations associated with azole resistance

The remaining 48 mutations detected across strains were not associated with the coding sequence of any gene, and thus their role in resistance is not clear (S4 Table). However, 22 of these SNPs were found in multiple isolates raising the intriguing possibility that those mutations do, in fact, play a role in resistance. Twenty-six mutations were found only in a single isolate, and could be a consequence of variability in genomic sequence not associated with drug resistance.

### Genetic recombination and isolation of drug resistance determinants

We took advantage of the mating ability of the isogenic pair to determine the association of particular mutations with drug resistance phenotype. To this end, we crossed the three highly azole-resistant isolates selected above from each intermediate resistance lineage from both parents (AFB62 and AFB62F9) in each drug with their drug sensitive mating partner (Fig 4B). The most fertile crosses, abundant cleistothecia with viable ascospores (>2000 fungal balls), were chosen for progeny sequencing (S5 Table). Ascospores from the selected crosses were grown on varying levels of the each azoles: no drug, intermediate (1 – 3X MIC), and high (10X) drug concentration (Fig 4B). The logic behind this approach is that mutations with no association to drug resistance will be equally present in the mating strains under no drug selection and any drug concentration, while those SNPs that confer resistance to the drug will be found more frequently under intermediate selection, and perhaps exclusively under high selection. Therefore, after selection, the genomes of the resulting pool of offspring were determined and the frequencies of each of the mutations from the resistant strains were tracked through each of the drug concentrations (no drug, 1X MIC, 10X MIC). As a control, we analyzed the frequency of *MAT1-1* and *MAT1-2* in the pool because these loci are not associated with resistance and would therefore be expected at a 1:1 frequency with or without drugs.

We found that all the *erg11A* gene mutations demonstrated the expected patterns in offspring pools (Table 2). In general, for each progeny under no selection, both the sensitive and resistant alleles could be detected in the population at similar frequencies (1: 1 ratio, or 0.5 / 0.5 allele frequency). In contrast, only the resistant allele was detected at either the intermediate or high drug concentrations in all posaconazole and voriconazole crosses (Table 2; allele frequency of 1). For voriconazole, the *hmg1* alleles followed the same patterns as the *erg11A* resistance alleles, showing that these mutations were also under voriconazole selection, and must therefore contribute to resistance. In contrast, the mating loci were usually found at a 1:1 ratio in each pool. No other mutations in voriconazole or posaconazole isolates could be associated with the resistance phenotype.

**Table 2:** Variant allele frequencies of relevant mutations in sequenced pools of mating offspring under no drug, intermediate, and high-level selection for each drug.

Strain^a^	Drug (concentration (μg/mL))	Hypothetical (promoter region)	*ganA* (L240Syn)	ABC transporter (promoter region)	*erg11* (G54R)	*erg11* (D261Syn)	*erg11* (G432Syn)	*hmg1* (H312Y)	*hmg1* (E307D)	*hmg1* (F311L)	*ssc70* (R317C)
AFB62I11	Itra (0)	1^b^	0.5	0.5	**1** ^**c**^						
AFB62I11	Itra (2)	1	0.5	0.5	**0.5**						
AFB62I11	Itra (10)	1	0.5	0.5	**1**						
AFB62F9I31	Itra (0)	1							**Ref**		
AFB62F9I31	Itra (2)	1							**1**		
AFB62F9I31	Itra (10)	1							**1**		
AFB62F9I22	Itra (0)	1									**Ref**
AFB62F9I22	Itra (2)	1									**1**
AFB62F9I22	Itra (10)	1									**1**
AFB62P13	Posa (0)	1	0.5	0.5	**0.5**						
AFB62P13	Posa (0.128)	1	0.5	0.5	**1**						
AFB62P13	Posa (1.3)	1	0.5	0.5	**1**						
AFB62P23	Posa (0)	1	0.5	0.5	**0.5**						
AFB62P23	Posa (0.128)	1	0.5	0.5	**1**						
AFB62P23	Posa (1.3)	1	0.5	0.5	**1**						
AFB62P31	Posa (0)	1	0.5	0.5	**0.5**						
AFB62P31	Posa (0.128)	1	0.5	0.5	**1**						
AFB62P31	Posa (1.3)	1	0.5	0.5	**1**						
AFB62V11	Vori (0)	1	0.5	0.5		**0.5**			**0.5**		
AFB62V11	Vori (0.376)	1	0.5	0.5		**0.5**			**1**		
AFB62V11	Vori (2)	1	0.5	0.5		**1**			**1**		
AFB62V33	Vori (0)	1	0.5	0.5			**0.5**			**0.5**	
AFB62V33	Vori (0.376)	1	0.5	0.5			**0.5**			**1**	
AFB62V33	Vori (2)	1	0.5	0.5			**1**			**1**	
AFB62F9V23	Vori (0)	1				**0.5**		**0.5**			
AFB62F9V23	Vori (0.376)	1				**0.5**		**1**			
AFB62F9V23	Vori (2)	1				**1**		**1**			

^a^ The listed resistant strain was mated with the isogenic sensitive strain of the opposite mating type. (e.g. AFB62I11 (ItrR) was mated with AFB62F9)

^b^ 1: represents a variant allele frequency of 1 (i.e. only the mutant SNP was found). 0.5: represents a 1:1 ratio of the reference and mutant alleles. Ref: signifies only the reference allele was detected in the pool.

^c^ Mutations associated with the resistance phenotype because they followed the expected pattern are highlighted in bold. All blank cells represent the reference allele and were left blank because those mutations were not relevant in a given cross.

Interestingly, the *erg11A* mutant alleles followed the same distribution when the itraconazole-resistant AFB62 parent was mated with the sensitive F9 derivative (Table 2). However, when the resistant F9 was crossed with the sensitive AFB62, the *erg11A* resistance allele could no longer be detected. Furthermore, two of the F9-itraconazole resistant strains did not harbor mutations in the *erg11A* or *hmg1* genes, suggesting that these strains had alternate mechanisms of resistance. The mutation in *ssc70* present in both of the high-resistant parents in these crosses also showed association with the drug resistance phenotype, suggesting that in these lineages, the *ssc70* mutation was sufficient to confer itraconazole-resistance. There were no other mutations that followed the expected pattern in these pools, thus showing that in addition to the two genes described for posaconazole and voriconazole (*erg11A* and *hmg1)*, *ssc70* also played an important role in azole resistance. Thus, from the original SNPs identified in any given resistant strain (up to 13) we could narrow down the specific resistance-conferring mutation to only 1 or 2 in any strain, and to a total of 5 genes across all strains.

In order to confirm the role of our newly identified mutations in azole resistance, we attempted to make mutations in *hmg1* and *ssc70* genes to recapitulate the phenotype. We succeeded in generating mutant *hmg1P1038T* which had the same mutation present in strain AFB62V3 and its high-level voriconazole resistant derivatives AFB62V31 and AFB62V33. Strain *hmg1P1038T* was resistant to 4X the MIC of the wild-type strain for voriconazole. We tested whether *hmg1P1038T* was also resistant to the other azoles. Indeed, the strain was more resistant to itraconazole with 4X higher MIC, and 2X MIC posaconazole resistance (Table 3). The MIC for strain AFB62V31, that had the *erg11* mutation in addition to the *hmg1* mutation, was even higher (16X) suggesting that the combination of mutations conferred high-level resistance to all azoles. The data also showed that the spontaneous mutant AFB62F9I22 carrying an *ssc70* mutation was more resistant to posaconazole and voriconazole in addition to itraconazole compared to the naïve AFB62F9 (Table 3). Despite multiple attempts to generate an *ssc70* mutant, we were not successful, suggesting that this gene may be essential and/or not be amenable to genetic manipulation. In fact, the *SSC1* gene in *S*. *cerevisae*, the ortholog of *A*. *fumigatus SSC70*, is known to be an essential gene [33].

**Table 3:** Azole resistance in spontaneous and engineered strains.

	MIC (μg/ml)		
Strain	Voriconazole	Posaconazole	Itraconazole
AFB62	1	0.25	1
AFB62*hmg1*P1038T	4	0.5	4
AFB62V31	>16	>16	>16
AFB62F9	1	0.25	1
AFB62F9I22	2	0.5	2

MIC determined by broth dilution method.

Combined, these results demonstrate that implementing whole-genome sequencing coupled with classical genetic approaches can identify genetic loci responsible for a particular phenotype with unparalleled detail.

## Discussion

The discovery of the *A*. *fumigatus* sexual cycle [4] opened a road to genetic studies by recombinational analysis in this pathogen. However, the six-month incubation period required for the completion of sexual reproduction hampered its wide use as a genetic tool. Our subsequent identification of a supermater pair which can complete sexual cycle in 4 weeks [5] made the use of sexual cycle more tractable in studies of the pathobiology of *A*. *fumigatus*. To further enhance the use of this mating pair as genetic tool, we generated a *MAT1-2* strain isogenic to AFB62 (*MAT1-1*) strain by successive backcrossing of *MAT1-2* progeny obtained in each generation to the foundational strain AFB62. The genome of a ninth generation *MAT1-2* progeny (AFB62F9) was found to be essentially identical to that of AFB62, except for the mating locus, the absence of a single 28Kb region, and 849SNP, thus providing a mating pair in which there is very little genetic diversity. The 28 Kb, which contains a reverse transcriptase and a bacteriodetes-like chitinase among others, appears to be unique to AFB62, as it is absent in 7 other sequenced strains of *A*. *fumigatus* [20]. We have backcrossed the strains until the 11^th^ generation to improve the degree of isogenicity even further. However, generations beyond F_9_ showed lower and an inconsistent degree of fertility which would be unsuitable as a genetic tool for recombination analysis. Since fertility of the AFB62 x AFB62F9 mating was comparable to the supermater pair AFB62 x AFIR928 [5], and the 28 Kb fragment is unique to AFB62, this 28 Kb sequence may play a role in its high fertility. The molecular dissection of the 28 Kb sequence is warranted to understand its role in fertility.

The sexual cycle in aspergilli requires an array of genes to ensure successful reproduction. A genome wide comparison of three aspergilli, *A*. *fumigatus* (strain AF293), *A*. *nidulans*, and *A*. *oryzae*, identified 140 orthologs that were implicated in the mating process of *Saccharomyces cereviseae* [2,34]. Genomic comparison between *A*. *fumigatus* AF293 (*MAT1-2*) and A1163 (*MAT1-1*) strains showed that A1163 strain possess all the genes found in the AF293, except for the genes Afu3g06160 and Afu3g06170 within the mating locus. The gene Afu3g06170 is known to encode a transcriptional factor with a High Mobility Group (HMG) domain. The gene Afu3g06160 is not fully characterized and thus, its role in the mating process is unknown. *MAT1-1* strains possess only one gene in the mating locus, a transcriptional factor (AFUB_04290) that has an alpha box domain. The proteins in the *MAT* loci are important not only for sexual identity but also they are likely to act as master regulators of the sexual cycle, since they are both transcriptional factors. In fact, Scewczyk and Krappmann [35] reported that the mating proteins of *A*. *fumigatus* regulate the expression of *ppga*, a pheromone-encoding gene, in a reciprocal manner: the *MAT-1* protein is required for *ppga* expression while the *MAT-2* protein appears to repress its expression.

The predicted function of some of the proteins encoded in the 400 Kb region suggests a possible involvement in reproduction, and consequently potentially being selected during mating. For instance, found in this region are genes encoding for a sister-chromatid segregation protein homolog (*src1*), an adenylate cyclase (*acyA*), and two transcription factors including TFIID. The SRC1 protein was shown to be important in chromatin organization during mitosis in the yeast *Saccharomyce cerevisiae*, thereby ensuring proper segregation of nucleic acid material during nuclear division [36,37] and ACYA has been implicated in sporulation and is central to intracellular signaling [38]. Our findings suggest the possibility that specific alleles that are important for mating regulation might not be equally represented/functional in the *MAT1-1* and *MAT1-2* mating type strains of *A*. *fumigatus* and that they likely reside outside of the mating type locus. Evolution of the *MAT* locus in aspergilli is somewhat controversial. One of the evolution models hypothesize that heterothallic species derived from a common homothallic ancestor whereas in another model homothallic species evolved from heterothallism [39,40]. The results of genome sequencing and comparison between AFB62 and AFB62F9 provide an opportunity to uncover the components required for normal genetic segregation.

Generation of isogenic strains and their use for genetic analysis enabled extensive progress in the study of molecular pathobiology of other fungi. For instance, the involvement of mating type in the virulence of *Cryptococcus neoformans* was assessed with using isogenic strains [6–9]. Since pathology of *A*. *fumigatus* has also been suggested to correlate with its mating type [10,11,41] the isogenic pair, AFB62 and AFB62F9, was used to test this correlation. In all of the models tested, hydrocortisone treated or CGD mice as well as *G*. *mellonella* larvae, the virulence of AFB62 (*MAT1-1*) and AFB62F9 (*MAT1-2*) was similar. Also, histological sections of lungs from mice infected with either AFB62 or AFB62F9 did not reveal substantial differences in the manner by which these strains caused the disease. In previous studies Alvarez-Perez and collaborators [10] described that among 28 strains isolated from IA cases, 22 were *MAT1-1* and only six were *MAT1-2* suggesting a correlation between *MAT1-1* and invasive aspergillosis. Furthermore, survival studies on larvae of *G*. *mellonella* showed a slightly higher virulence of *MAT1-1* compared to *MAT1-2* in the tested clinical isolates [11]. Since the pathology of *A*. *fumigatus* involves numerous factors, assessing the specific contribution of the *MAT* locus in virulence of strains is not possible unless the strains are congenic except for the *MAT* locus. Although predominance of a single mating type in clinical cases has been shown in other fungi, for instance *Histoplasma capsulatum* [42–44] and *Cryptococcus neoformans* [6,9], it is not the case with *A*. *fumigatus* and our findings suggest that the presence of either *MAT1-1* or *MAT1-2* locus in the genome of *A*. *fumigatus* does not predispose the fungus to higher virulence or affect the development of IA in murine models.

Azoles are the most frequently used drugs in treating IA. As a consequence, the incidence of resistance to azoles is also on the rise. Currently the most commonly reported mechanism conferring azole resistance in *A*. *fumigatus* is mutation in the *erg11A* (*cyp51A*) gene, leading to alterations in the target protein. The most common resistance-conferring mutation in this gene is an alteration at codon 98 (L98H), alone or in combination with a tandem repeat in the promoter region. Other variations are frequently detected in this gene, many of which have been demonstrated to confer resistance to azoles [12]. In this study we not only identified single mutations with a dominant phenotype like those affecting *erg11A*, but also other associated mutations that form part of the complex phenotype of resistance. Particularly, we found mutations in the HMG CoA reductase (*hmg1)* gene associated with voriconazole resistance, but no other azole. Voriconazole has a significantly different chemical structure than itraconazole and posaconazole [13] which might explain the difference in distribution of mutations. It is likely that voriconazole targets both the 14-alpha sterol demethylase and HMG CoA reductase enzymes and consequently both mutations are necessary for full resistance. Indeed, we found that the strains containing mutations in both *ERG11* and HMG CoA reductase (like AFB62V31) were much more resistant to voriconazole than the HMG CoA reductase mutant alone. All the mutations we identified in HMG CoA reductase occurred within the sterol sensing domain that is responsible for feedback regulation of sterol-regulated genes and sterol biosynthesis [32]. Mutations in this domain in other species can result in proteins that are highly stable and unable to respond to sterol degradation products [45]. If the same is true in *A*. *fumigatus*, the *hmg1* mutants should also be resistant to other azoles. We found that the spontaneous and our engineered *hmg1P1038T* mutant were resistant to all three azoles at roughly the same levels (2X – 4X MIC than wild-type). The results suggest that these mutant strains are indeed less sensitive to toxic intermediates that result from azole inhibition of ergosterol biosynthesis in general. However, the underlying reasons for *hmg1* mutations being so frequently identified under voriconazole selection and not detected at all under itraconazole or posaconazole selection are not clear.

We found mutations in the heat shock protein SSC70 under itraconazole pressure which followed the expected segregation pattern in sexual crosses. Heat shock proteins, like Hsp90, and the stress response in general, have been associated with azole and echinocandin resistance in *Aspergillus fumigatus* [46,47]. The *ssc70* transcript has been shown to be differentially regulated in response to stress and antifungals [48,49], though specific mutations have not been shown as potential resistance mechanisms. The mutation we identified resulted in a substitution of an arginine to cysteine residue in the nucleotide exchange factor domain of SSC70. The specific mechanism that leads to resistance is not clear, but may be a result of increased turnover during nucleotide exchange, thus leading to a higher capability of dealing with misfolded proteins as a consequence of sterol toxic byproduct accumulation. We were unable to engineer an *ssc70* mutant strain, which suggests that the balance between turnaround and toxic byproducts is essential for cell development in *A*. *fumigatus*.

Lastly, we also found a mutation in *erg25* (C-4 sterol methyl oxidase) in a highly itraconazole-resistant isolate. The mutation results in an altered HXHH motif that affects the coordination of the di-iron cluster that functions in the reaction center of the oxidase. The enzyme ERG25 acts downstream of ERG11, and mutations in this gene have been commonly associated with suppression of *erg11A* mutations. For example, in a recent study to identify *A*. *fumigatus* mutations associated with azole resistance, Camps *et al*. [19] also found mutations in *erg25*, though they did not find that the mutations in this gene alone could result in azole resistance. In their sexual crossings, the *erg25* mutation was present in both susceptible and resistant strains and their attempt of gene replacement in a susceptible background did not result in any itraconazole-resistant strains. It is noteworthy, however, that both studies identified mutations in the same gene. In our study, we detected a mutation that directly changed the active site of the enzyme, while Camps *et al*. found a truncating mutation [19]. The sexual crossings in our study between F9I11 (containing the *erg25* mutation) and the susceptible AFB62 parent resulted in modest numbers of ascospores at all levels of itraconazole. We were not able to sequence the pools from this cross due to limited DNA yield from resulting pools. Thus, while it was not possible to observe the specific selection of *erg25* mutations after sexual crossing and recombination, further clarification of its involvement is warranted. In *Saccharomyces cerevisiae* it was shown that the lanosterol and ergosterol biosynthetic pathways are intimately intertwined and that in the absence of ergosterol (either because of azoles or mutation), the cells are still viable, presumably because of the incorporation of lanosterol or other sterols in the membrane instead of ergosterol [50]. We also found a high frequency of mutations in *ganA—*that encodes an ABC multi-drug transporter, a hypothetical protein, and a non-coding SNP, which almost always appeared together in the absence of other mutations in *erg11A* or *hmg1*. In sexual crosses these mutations did not follow the expected differences in frequency had they been linked with resistance. However, this might have been a result of our study design, rather than a lack of significance of these mutations in azole resistance. We purposefully selected only the high-level resistant isolates for the sexual crossing experiments with the hypothesis that several mechanism of resistance would be at play at this high level, and thus growing the progeny under varying amounts of drug would elucidate those mechanisms. We found, however, that mutations in *erg11A* and *hmg1* alone were sufficient for high-level resistance, and as a consequence all other mutant alleles could have followed random segregation in their presence. Application of sexual crossing to strains with non-dominant mutations in *erg11A* or *hmg1* would help clarify the role of these mutations. Similarly, it was possible that new *de novo* mutations would occur in a subpopulation of the pools upon drug selection of the pool. In fact, based on our initial selection in which we plated 10^5^ conidia, we would expect to see up to 10^2^ *de novo* azole resistant strains. This mutant abundance translates to an allele frequency of 0.001, which is below the level of detection for our bulk sequencing analysis. Thus, our strategy focused on already identified mutations in an attempt to clearly demonstrate the role of specific mutations in resistance.

The mating efficiency and isogenicity of AFB62F9 and AFB62 make these strains useful for recombinational analysis in genetic studies of *A*. *fumigatus* pathobiology, particularly for traits for which selection of a phenotype, such as drug resistance, can be accomplished. In this study we demonstrate that classical genetic experiments coupled with whole genome sequencing is a highly effective way of identifying mutations associated with a specific phenotype in *A*. *fumigatus*.

## Methods

### Strains

The strains AFB62 (*MAT1-1*) and AFIR928 (*MAT1-2*) of *A*. *fumigatus* [5] were employed as the parental strains in the construction of an isogenic pair. The strains AFB62*Δabr2* [5] bearing brown conidia and AFB62F9*Δalb1* bearing white/albino conidia were used for genetic recombination assays.

### Genomic transformation

AFB62F9*Δalb1*: deletion of the gene *alb1*/*pksP* gene (Afu2g17600) was obtained by transforming AFB62F9 with the vector pDHt/*alb1*::*hph* via ATMT [51]. Strain *AFB62hmg1P1038T*: a construct harboring the P1038T mutation in the *hmg1* gene of AFB62V31 was created to replace the native *hmg1* locus in AFB62. The construct harbored the hygromycin resistance cassette flanked by a 3,870 bp fragment consisting of the *hmg1* (Afu2g03700) ORF plus 300 bp sequence adjacent to the termination code of the gene on one side and a 1,002 bp fragment located immediately after the termination code of the gene on the other side. Thus, the 1,002 bp fragment has the repeat of the 300 bp adjacent to the termination code cloned into the 3,870 bp fragment. Both fragments were amplified from AFB62V31. The construct was cloned into the pDHt/SK vector and electroporated into *Agrobacterium tumefaciens* to transform AFB62 conidia [51]. Southern analysis confirmed homologous recombination of a single copy of the construct in the mutant *AFB62hmg1P1038T*. Sequencing confirmed the P1038Thr mutation.

### Mating and progeny analysis

Mating, progeny isolation and viability were carried out as described elsewhere [5]. Generation of isogenic set: F1 progeny, which was produced by mating of AFB62 with AFIR928 [5], was backcrossed with AFB62. Ascospores from one of the most fertile crosses between F1 and AFB62 were isolated and backcrossed with AFB62 to generate F2 progeny. Back-crossings were successively repeated until F9 generation was obtained. Each backcrossing was carried out with 10–20 progenies isolated from the most fertile crosses from each generation. Mating plates were analyzed at 4–8 weeks. Heat-treated ascospores (70°C for 30 min) were plated on either malt extract agar (MEA) or *Aspergillus* minimal media (AMM) agar (with or without 10 mM of uracil and 10 mM of uridine), incubated at 37°C for 48h and stored for further use. Controls for conidial killing were carried out with parental conidia harvested from mating plates. Conidia were heat-treated at 70°C for 30 min, plated on MEA and incubated at 37°C. After 48 h no growth was observed confirming that parental conidia were killed when incubated at 70°C for 30 min. Mating type of progeny was assessed by PCR with primers specific for amplification of *MAT1-1* and *MAT1-2* mating types [5]. Mating of azole resistant isolates: the AFB62 resistant isolates were mated with the naïve AFB62F9 strain and the AFB62F9 resistant isolates were mated with the naïve AFB62. Crosses were analyzed at 20 to 25 weeks. Ascospores from fertile crosses were heat-treated, inoculated on MEB containing various drug concentrations and incubated for 48–72 h at 37°C and 220 RPM. Progeny of voriconazole resistant isolates were grown on 0 (no drug), 0.376 (1.5X MIC) and 3 μg/ml (12X MIC) of voriconazole. Progeny of itraconazole resistant isolates were grown on 0 (no drug), 10 (5X MIC) and 100 μg/ml (50X MIC) of itraconazole. Progeny of posaconazole resistant isolates were grown on 0 (no drug), 0.128 (1X MIC), 1.3 (AFB62F9 derivatives, 10X MIC) and 10 μg/ml (AFB62, 10X MIC) of the drug. Fungal ball-colonies derived from ascospores germination were washed with PBS, counted and lyophilized. Pools ranging from 100–2000 fungal-ball colonies derived from each cross were used for DNA isolation with DNeasy Plant Kit (Qiagen) according to manufacturer’s instruction.

### DNA isolation

1x10^7^ conidia were inoculated into 10 ml of yeast nitrogen broth (BD, Sparks, MD) and incubated for 24 hours with shaking at 200 rpm. Mycelia were harvested, lyophilized and DNA isolated with DNeasy Plant Kit (Qiagen) according to manufacturer’s protocol. DNA quality was monitored with 0.8% agarose gel electrophoresis.

### Comparative genomic hybridization (CGH)

CGH was performed as described previously [2]. Hybridizations of the biological replicates were repeated in dye-swap sets. Hybridized slides were scanned and analyzed to obtain relative hybridization profiles. Normalized data were averaged over replications, and genes with differential hybridizations at the 95% confidence level were determined using intensity-dependent *Z*-scores (with *Z* = 1.96). The resulting data were analyzed to identify genes variably present in any of the probed genomes. Any genes with hybridization signals with greater than 1.4 fold differences (-0.75 ≤ log2 ≤ 0.75) between two different strains were considered absent or diverged.

### Genome sequencing of AFB62 and AFB62F9

Genomic DNA was sequenced using paired-end Illumina GA-II to 44-fold coverage. The assembly of AFB62 was done with CLC Genomics Workbench 4.1 using strict parameters: length fraction = 0.9, similarity = 0.9, allowing for random placing of non-specific reads. Sequence data for this project has been deposited to NCBI (BioProject ID: PRJNA237785).

### Virulence

Mice with Chronic Granulomatous Disease (CGD) and mice immunosuppressed with hydrocortisone were used for murine model studies. CGD mice, strain gp91^*phox*^-deficient mice (B6.129S6-*Cybb* ^*tm1Din*^ */J*) (The Jackson Laboratory), were inoculated with 30 μl of 3.33 x 10^5^ conidia/ml (5 mice per group). The immunosuppressed model was carried out with BALB/c mice (National Cancer Institute Division of Cancer Treatment, Bethesda, MD) treated with hydrocortisone acetate (Sigma, St Louis, MO) as described elsewhere [5]. These mice received 30 μl of 3.33 x 10^7^ conidia/ml (10 mice per group). All mice were inoculated via pharyngeal aspiration [52]. Lung sections were stained with hematoxylin and eosin (H&E) and Gomori methenamine silver (GMS) for histopathology analysis. Survival data were analyzed using the log rank test. The animal experiments were performed under protocol approved by IACUC of the National Institute of Allergy and Infectious Diseases at the U. S. National Institutes of Health (NIH). *Galleria mellonella* larvae in the final larval stage (Vanderhorst Wholesale, Inc., St. Marys, OH) were inoculated with 5 μl of 2x10^7^ conidia/ml as described elsewhere (15 larvae per group) [24,53]. Larvae were then incubated at 37°C and mortality monitored daily. Mortality was assigned based on change of color from light beige (healthy larvae) to dark brown and unresponsiveness to tactile stimulus.

### Ethics statement

The Institutional Animal Care and Use Committee of the National Institute of Allergy and Infectious Diseases approved all animal studies (#A4149-01). Studies were performed in accordance with recommendations of the Guide for the Care and Use of Laboratory Animals of the National Institutes of Health.

### Minimal inhibitory concentration (MIC)

MIC assays were carried out following the method for broth dilution [54] or with Etest. Etest was performed by following recommended protocols (BioMerieux). Briefly, 10^6^ conidia were plated on MEA plates, the Etest strip was placed on the surface of the plate and the plates were incubated at 37° for 6 days. The MIC was determined as the concentration in the Etest strip where the growth was inhibited. Unless otherwise specified, MICs were determined by Etest.

### Selection and culture of drug resistant isolates

1x 10^5^ conidia of AFB62 or AFB62F9 were plated on MEA containing sub-MIC concentrations of itraconazole (2.0 μg /ml), posaconazole (0.032 μg/ml), or voriconazole (0.094 μg/ml) and incubated at 37°C for 1 week. Resulting conidia were resuspended in 0.01% TritonX-100 and 1x 10^5^ conidia were plated on 1X – 3X MIC (S3 Table) in order to obtain fewer than 100 colonies per plate. Three resistant colonies from each azole for each parent (AFB62 or AFB62F9) were picked, grown individually in MEA plates containing the same amount of azole as was used for selection, and incubated until conidia were visible, these represent the intermediate resistance isolates. A total of 18 isolates were collected at this stage (2 parents x 3 drugs x 3 isolates each). 1x 10^5^ conidia from the resistant strains were plated onto MEA containing 10X – 50X MIC in order to obtain fewer than 100 colonies on each plate. Again, three colonies from each plate were picked and grown individually on MEA containing the high MIC concentration of the corresponding azole (S3 Table). A total of 48 highly resistant isolates were collected (2 parents x 3 drugs x 3 lineages from intermediate selection plates x 3 isolates) and stored as conidial stocks.

For DNA isolation of intermediate or highly resistant isolates, 1x 10^7^ conidia were inoculated into 10 ml of malt extract broth (MEB) containing drug in various concentrations. Drug resistant isolates of AFB62 and AFB62F9 were grown on voriconazole at concentrations of either 0.376 or 2 μg/ml or itraconazole at either 2 or 10–50 μg/ml. AFB62 posaconazole isolates were grown on either 0.32 or 10 μg/ml and AFB62F9 isolates were grown on either 0.128 or 1.3 μg/ml of posaconazole. Mycelia were harvested after 24–48 h incubation at 37°C in orbital shaker (220 rpm). DNA was isolated using DNeasy Plant Kit (Qiagen) according to manufacturer’s instruction. DNA quality was monitored by 0.8% agarose gel electrophoresis.

### SNP detection and analysis

For intermediate and high-resistance isolates obtained during *in vitro* selection, the sequencing reads from each isolate were first trimmed on quality (q> = 30) and then mapped to the AFB62 assembly using mpileup using default parameters [55]. Identified single nucleotide polymorphisms (SNPs) were filtered to identify high quality SNPs using the following criteria: AC = 1, AF = 2, DP > = 10, MQ> = 40 and FQ< = -35, and covered by at least 4 reads in each direction. For pools, the proportion of reads with the wild-type and mutant nucleotides were queried from the unfiltered mpileup results of mapping the reads from the pools to the AFB62 genome.

## Supporting Information

S1 Fig Progeny from *Aspergillus fumigatus* isogenic pair. Ascospores isolated from a cross between an AFB62 mutant producing brown conidia (AFB62*Δabr2*) and an AFB62F9 mutant producing white conidia (AFB62F9Δ*alb1*). Plate shows progeny with white, brown and green (arrows) conidia.
(TIF)

S2 Fig Chromosomal fragment containing the *MAT1-1* locus and lacking 28 Kb inherited from AFIR928. Close-up of AFB62F9 reads mapped to the AFB62 region ~200Kb downstream of the *MAT1-2* locus showing the missing 28Kb fragment. Top track: average coverage of the genome was 48X. The 28 Kb locus had no mapped reads so the coverage drops to 0X. Numbers refer to coordinates on contig613 in the AFB62 assembly (coordinates 1635139–1864259 on chromosome III of AF293). Second track: SNP density per 1,000 bp is depicted in red rectangles. Bottom tracks: annotated protein-coding genes in the AFB62 assembly. Arrows depict the coding strand.
(TIF)

S1 Table Comparative genome hybridization (CGH) results showing only 7 gene divergence between AFB62F4 and AFB62. (XLSX)

S2 Table AFB62 unique genes when compared to AF293 and A1163. (XLSX)

S3 Table Minimum inhibitory concentrations (Etest) of AFB62 or AFB62F9 to select antifungals. (XLSX)

S4 Table Summary of all identified SNPs in intermediate and high level resistant isolates. (XLSX)

S5 Table Fertility of azole resistant and sensitive isogenic strains. (DOCX)
