# Supplementary figures and images for: Genetic Analysis Using an Isogenic Mating Pair of Aspergillus fumigatus Identifies Azole Resistance Genes and Lack of MAT Locus’s Role in Virulence
Source: PLoS Pathog. 2015 Apr 24;11(4):e1004834. doi: 10.1371/journal.ppat.1004834 (PMC4409388; doi:10.1371/journal.ppat.1004834)

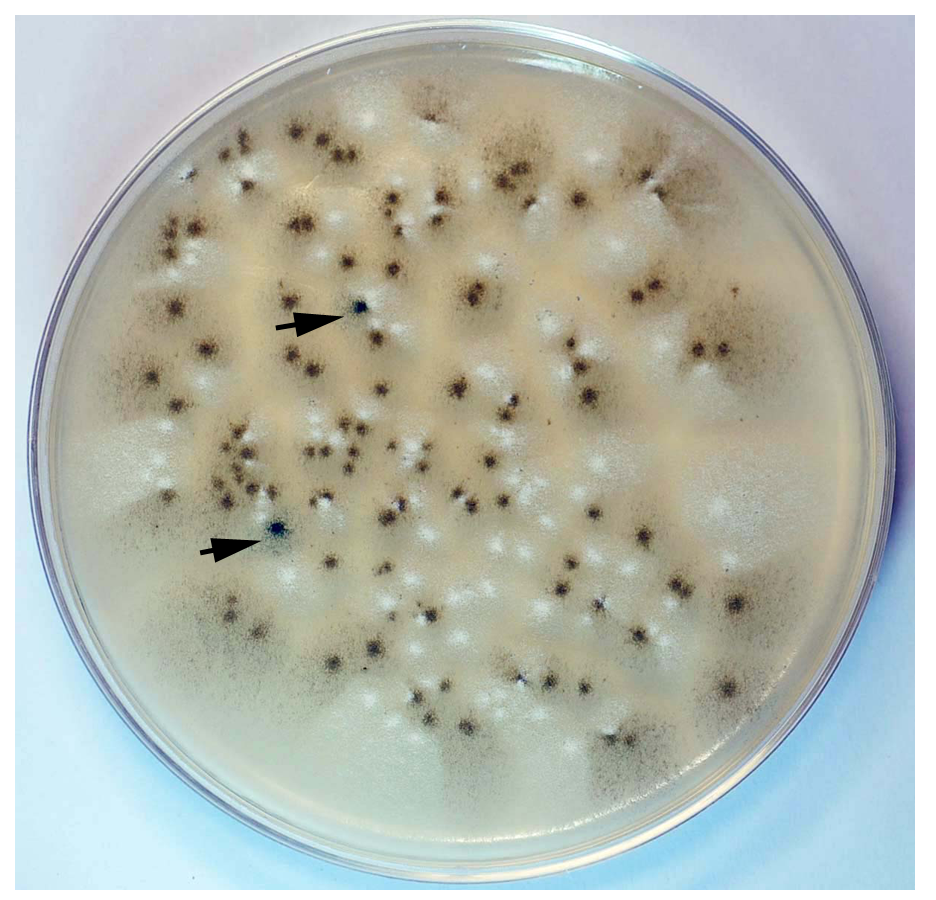

Supplement: S1 Fig — Ascospores isolated from a cross between an AFB62 mutant producing brown conidia (AFB62Δabr2) and an AFB62F9 mutant producing white conidia (AFB62F9Δalb1). Plate shows progeny with white, brown and green (arrows) conidia. (TIF) [file ppat.1004834.s001.tif]

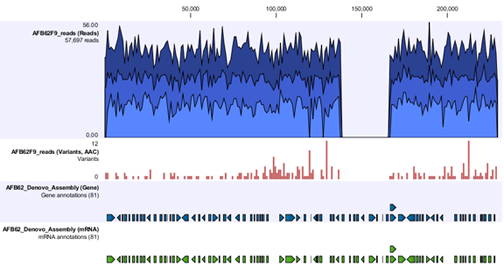

Supplement: S2 Fig — Close-up of AFB62F9 reads mapped to the AFB62 region ~200Kb downstream of the MAT1-2 locus showing the missing 28Kb fragment. Top track: average coverage of the genome was 48X. The 28 Kb locus had no mapped reads so the coverage drops to 0X. Numbers refer to coordinates on contig613 in the AFB62 assembly (coordinates 1635139–1864259 on chromosome III of AF293). Second track: SNP density per 1,000 bp is depicted in red rectangles. Bottom tracks: annotated protein-coding genes in the AFB62 assembly. Arrows depict the coding strand. (TIF) [file ppat.1004834.s002.tif]
