# Supplementary material for: Genetic Analysis Using an Isogenic Mating Pair of Aspergillus fumigatus Identifies Azole Resistance Genes and Lack of MAT Locus’s Role in Virulence
Source: PLoS Pathog. 2015 Apr 24;11(4):e1004834. doi: 10.1371/journal.ppat.1004834 (PMC4409388; doi:10.1371/journal.ppat.1004834)
Supplement: S5 Table — (DOCX) [file ppat.1004834.s007.docx]

**S5 Fertility of azole resistant and sensitive isogenic strains.**

| Resistant isolate^a^ | Drug | Cleistothecia | Ascospores |
| --- | --- | --- | --- |
|  |  |  |  |
| AFB62P11 | **Posaconazole** | **+++** | **+** |
| AFB62P12 | **Posaconazole** | **+++** | **ND** |
| AFB62P13 | **Posaconazole** | **+++** | **+^*^** |
| AFB62P21 | **Posaconazole** | **++** | **ND** |
| AFB62P22 | **Posaconazole** | **+++** | **ND** |
| AFB62P23 | **Posaconazole** | **+++** | **+^*^** |
| AFB62P31 | **Posaconazole** | **+++** | **+^*^** |
| AFB62P32 | **Posaconazole** | **+** | **ND** |
| AFB62P33 | **Posaconazole** | **++** | **ND** |
| AFB62V11 | **Voriconazole** | **++** | **+^*^** |
| AFB62V12 | **Voriconazole** | **++** | **ND** |
| AFB62V13 | **Voriconazole** | **-** | **-** |
| AFB62V21 | **Voriconazole** | **+** | **+** |
| AFB62V22 | **Voriconazole** | **-** | **-** |
| AFB62V23 | **Voriconazole** | **+** | **-** |
| AFB62V31 | **Voriconazole** | **+** | **-** |
| AFB62V32 | **Voriconazole** | **-** | **-** |
| AFB62V33 | **Voriconazole** | **+** | **+^*^** |
| AFB62I11 | **Itraconazole** | **+++** | **+^*^** |
| AFB62I12 | **Itraconazole** | **-** | **-** |
| AFB62I13 | **Itraconazole** | **+++** | **+** |
| AFB62I21 | **Itraconazole** | **+++** | **+** |
| AFB62I22 | **Itraconazole** | **+++** | **+** |
| AFB62I23 | **Itraconazole** | **+++** | **+^*^** |
| AFB62I31 | **Itraconazole** | **++** | **+** |
| AFB62I32 | **Itraconazole** | **-** | **-** |
| AFB62I33 | **Itraconazole** | **+** | **-** |
| AFB62F9V11 | **Voriconazole** | **+** | **-** |
| AFB62F9V12 | **Voriconazole** | **+** | **+** |
| AFB62F9V13 | **Voriconazole** | **++** | **+** |
| AFB62F9V21 | **Voriconazole** | **++** | **+^*^** |
| AFB62F9V22 | **Voriconazole** | **++** | **ND** |
| AFB62F9V23 | **Voriconazole** | **++** | **+^*^** |
| AFB62F9V31 | **Voriconazole** | **+** | **ND** |
| AFB62F9V32 | **Voriconazole** | **-** | **-** |
| AFB62F9V33 | **Voriconazole** | **++** | **-** |
| AFB62F9I11 | **Itraconazole** | **+** | **+** |
| AFB62F9I12 | **Itraconazole** | **-** | **-** |
| AFB62F9I13 | **Itraconazole** | **++** | **+^*^** |
| AFB62F9I21 | **Itraconazole** | **++** | **+** |
| AFB62F9I22 | **Itraconazole** | **++** | **+^*^** |
| AFB62F9I23 | **Itraconazole** | **+** | **+** |
| AFB62F9I31 | **Itraconazole** | **++** | **+^*^** |
| AFB62F9I32 | **Itraconazole** | **-** | **-** |
| AFB62F9I33 | **Itraconazole** | **++** | **-** |

**^a^** AFB62 resistant isolates were mated with the naïve AFB62F9 strain and the AFB62F9 resistant isolates were mated with the naïve AFB62.

^b^ Plus signs indicate production of cleistothecia; increasing number of signs signifying greater abundances with +++ being >100. Negative signs mean there were no observed cleistothecia or ascospores. ND – not determined.

**^*^** The conidia from these progeny were pooled and sequenced for downstream analysis.
